# Reduced splenic volume and advanced age predict sepsis in patients with Howell–Jolly bodies: a retrospective cohort study

**DOI:** 10.1007/s12185-025-04050-9

**Published:** 2025-08-12

**Authors:** Kaori Uchino, Yuya Nakagami, Megumi Enomoto, Nozomi Shimizu, Kenichi Kondo, Takahiro Yamamoto, Yukie Sugita, Yuto Isaji, Sakura Saigusa, Yusuke Iida, Saki Shinohara, Tomohiro Horio, Satsuki Murakami, Shohei Mizuno, Kazuhiro Ikegame, Ichiro Hanamura, Akiyoshi Takami

**Affiliations:** 1 Division of Hematology, Department of Internal Medicine, Aichi Medical University School of Medicine, 1-1 Yazakokarimata, Nagakute, 480-1195 Japan; 2 Hematopoietic Cell Transplantation Center, Aichi Medical University Hospital, Nagakute, Japan; 3 Department of Clinical Laboratory, Aichi Medical University Hospital, Nagakute, Aichi Japan; 4 Central Department of Radiology, Aichi Medical University Hospital, Nagakute, Aichi Japan; 5 Department of Radiology, Aichi Medical University School of Medicine, Nagakute, Japan

**Keywords:** Functional hyposplenism, Howell–Jolly bodies, Splenic volume, Sepsis, Infection risk

## Abstract

Functional hyposplenism, defined as impaired splenic function in the absence of splenectomy, increases susceptibility to life-threatening infections. Although Howell–Jolly bodies (HJBs) are well-established markers for this condition, the predictive value of spleen volume for infection risk remains unclear. We retrospectively analyzed 95 non-splenectomized patients with HJBs from 2014 to 2024. We measured spleen volume by computed tomography and compared results with ideal values. We evaluated the associations between splenic volume and infections using univariate and multivariate logistic regression analyses. The median patient age was 66 years (range, 16−95); 72% were male. The median spleen volume was 34 mL, lower than the ideal median of 210 mL. Forty-eight percent of patients experienced at least one infection. Univariate analysis identified age ≥ 65 years and spleen volume < 34 mL as significantly associated with sepsis. Both factors remained independent predictors in multivariate analysis (age ≥ 65: odds ratio [OR], *p* = 0.039; spleen volume < 34 mL: OR 3.0, *p* = 0.047). Age ≥ 65 also predicted any infection (OR 3.1, *p* = 0.013), while low spleen volume demonstrated a trend toward significance (OR 2.2, *p* = 0.064). In non-splenectomized patients with HJBs, reduced spleen volume and older age independently increase susceptibility to sepsis. Computed tomography-based measurements may help identify functional hyposplenism and guide targeted prophylactic measures.

## Introduction

Functional hyposplenism is characterized by impaired splenic function in individuals without prior splenectomy. This condition increases susceptibility to severe, occasionally fatal infections, especially by encapsulated bacteria such as *Streptococcus pneumoniae*, *Haemophilus influenzae* type b, and *Neisseria meningitidis* [1, 2]. Functional hyposplenism can result from diverse etiologies, including congenital abnormalities, gastrointestinal and autoimmune disorders, hematologic and non-hematologic malignancies, infections, and immunosuppressive therapies [3–6]. Moreover, aging, malignancy, and certain molecular-targeted therapies have emerged as contributing factors to functional hyposplenism.

Despite its clinical relevance, this condition is frequently underrecognized. Early detection is crucial for initiating preventive strategies, including appropriate vaccinations and prophylactic antibiotic administration [7, 8]. Under normal conditions, the spleen removes nuclear remnants from circulating erythrocytes without destroying the cells. When splenic function is impaired, Howell–Jolly bodies (HJBs)—basophilic nuclear inclusions—become detectable in peripheral blood smears [1, 3, 5–7, 9–11].

Previous findings [6] indicate that HJBs detection serves as a screening tool for suspected functional hyposplenism and that splenic volumes in these patients are frequently below ideal levels. However, whether reduced spleen volume directly contributes to an increased risk of infection or influences infection outcomes remain unclear. Therefore, this study aims to clarify the relationship between spleen volume and infection incidence in non-splenectomized patients with HJBs. We hypothesized that computed tomography (CT)-based assessment of spleen volume could aid in diagnosing functional hyposplenism and identifying the need for intensified preventive strategies in at-risk individuals.

## Materials and methods

Following approval from the Institutional Review Board of Aichi Medical University School of Medicine (2023-239), patients with HJBs detected in peripheral blood smears via a standard optical microscope at Aichi Medical University Hospital from January 2014 to September 2024 were identified. HJBs were considered present when at least one red blood cell containing an HJB was identified in a single microscopic field at × 400 magnification. Patients aged ≤ 15 years or those lacking available computed tomography (CT) imaging were excluded. Retrospective data collection included underlying conditions, infections, and splenic volume measured on CT. Cases of sepsis, pneumonia, and urinary tract infections (UTIs) were included if confirmed via positive culture results and subsequently treated. Splenic volumes were quantified using CT imaging and analyzed with ZIOSTATION2 software (Ziosoft Inc., Tokyo, Japan). Ideal splenic volume was calculated using the following formula: 6.47 × body weight × age^−0.31^ (mL) [12]. The relative difference between measured and ideal splenic volumes was calculated as a percentage using the following formula: (the measured splenic volume -the ideal splenic volume)/the ideal splenic volume (%). Fisher’s exact test and logistic regression analysis were conducted to identify infection risk factors using the EZR software package for all analyses [13].

## Results

### Patient characteristics

**Fig. 1:**
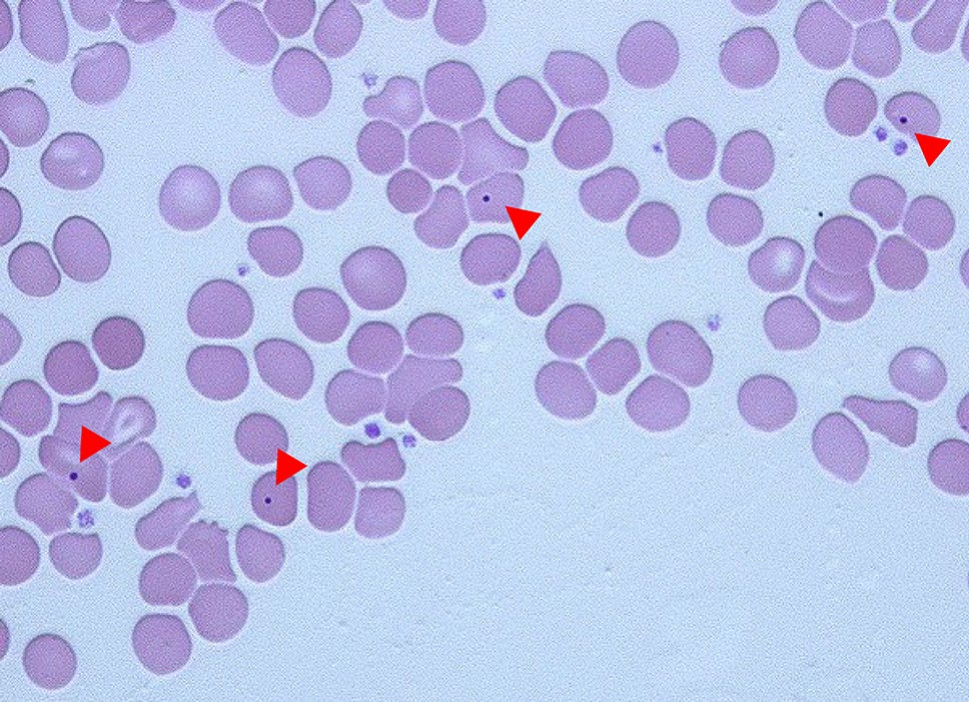
Peripheral blood smear of a representative patient showing HJBs indicated by red arrows (Wright–Giemsa stain; total magnification, × 400). *HJB* Howell–Jolly body

**Fig. 2:**
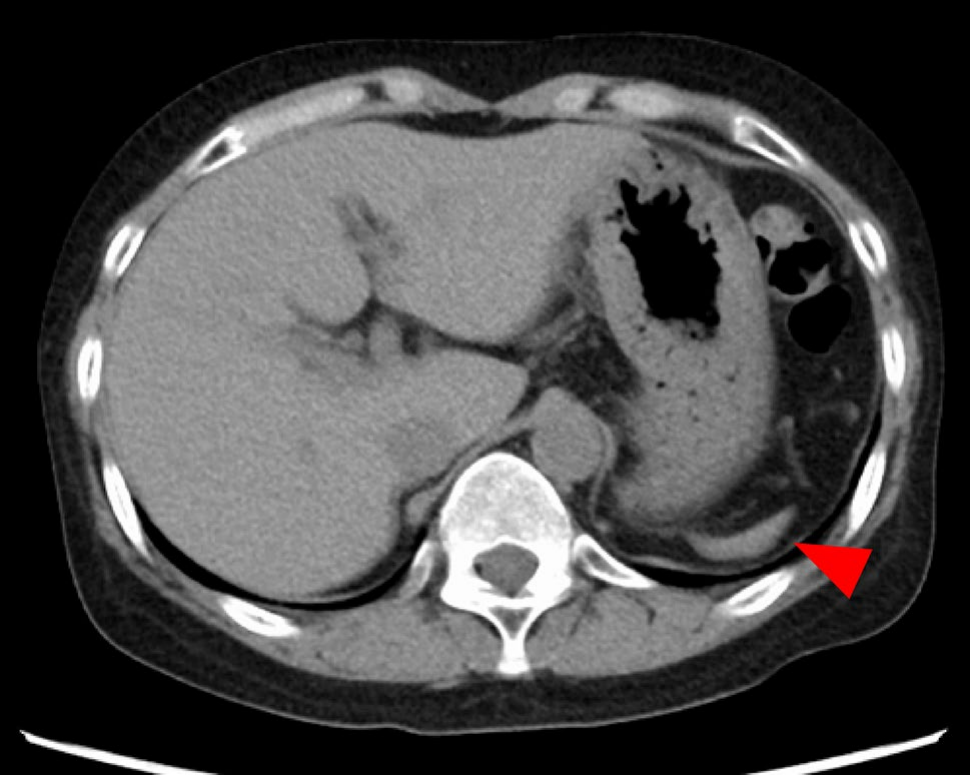
Abdominal CT image of a representative patient demonstrating marked splenic atrophy (red arrow). The measured splenic volume was 5.9 mL. *CT* computed tomography

**Fig. 3:**
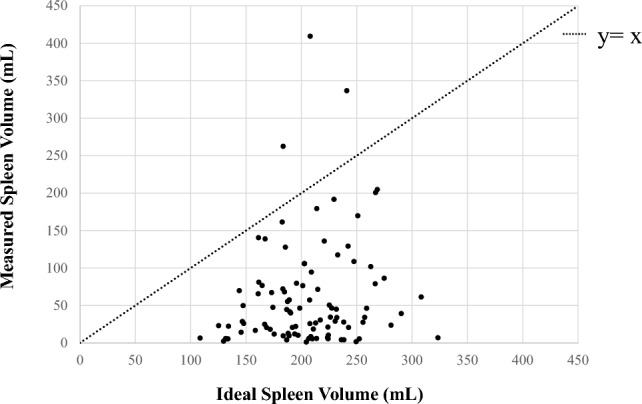
Measured vs. ideal spleen volume. Scatter plot comparing measured spleen volumes and ideal spleen volumes in 95 patients. Each dot represents one patient. The dashed line (*y* = *x*) indicates equality between measured and ideal volumes. Most patients fall below the line, indicating reduced spleen volume relative to ideal

Overall, 95 patients with HJBs who had not undergone splenectomy were included in this study. A representative peripheral blood smear demonstrating the presence of HJBs is shown in Fig. 1. The median age was 66 years (range, 16–95), with 68 (72%) being male. The median spleen volume was 34 mL (range, 1.2–410 mL), while the median ideal spleen volume was 210 mL (range, 110–320 mL). A representative CT image of a patient with marked splenic atrophy (measured volume, 5.9 mL) is shown in Fig. 2. In most cases, the measured spleen volume was smaller than the ideal spleen volume, with a median relative difference of –82% (range, –99 to 97%) (Fig. 3).

The most common underlying condition was cancer affecting 35 patients (37%), followed by alcoholic liver disease (12 patients, 13%), malignant hematological diseases (8 patients, 8.4%), post-hematopoietic stem cell transplantation (5 patients, 5.3%), and splenic ischemia (5 patients, 5.3%). Other conditions included inflammatory bowel disease, benign hematological diseases, abdominal aortic aneurysm (each in 3 patients, 3.2%), collagen diseases (2 patients, 2.1%), and various other conditions (19 patients, 20%) (Table 1).

**Table 1:** Characteristics of patients with HJBs excluding splenectomized patients

Variable	Value		Variable	Value
Number of cases	95		Underlying condition diseases, *n* (%)	
Patients age, years, median (range)	66 (16–95)		Cancer	35 (37)
Patient sex, *n* (%)			Alcoholic liver diseases	12 (13)
Male	68 (72)		Malignant hematological diseases	8 (8.4)
Female	27 (28)		Post hematopoietic stem cell transplantation	5 (5.3)
Volume of spleen, ml, median (range)	34 (1.2–410)		Splenic ischemia	5 (5.3)
Volume of ideal spleen, ml, median (range)	210 (110–320)		Inflammatory bowel diseases	3 (3.2)
The relative difference between the measured and ideal splenic volumes, %, median (range)	−82 (−99 to 97)		Benign hematological diseases	3 (3.2)
			Abdominal aortic aneurysm	3 (3.2)
Sepsis, *n* (%)	20 (21)		Collagen diseases	2 (2.1)
Pneumonia, *n* (%)	24 (25)		Others	19 (20)
Urinary tract infection, *n* (%)	19 (20)			
At least one infection, *n* (%)	46 (48)			

*HJBs* Howell–Jolly bodies

### Infectious characteristics

During the study period, 20 patients (21%) developed sepsis, 24 (25%) had pneumonia, and 19 (20%) experienced UTIs. Overall, 46 patients (48%) developed at least one of these infections—pneumonia, UTI, or sepsis. Among all infections, *Klebsiella pneumoniae—*an encapsulated bacterium—was the most frequently identified pathogen (Tables 2, 3, and 4).

**Table 2:** Causative pathogens of sepsis

Variable	Value	Variable	Value
*Klebsiella pneumoniae*	6	*Veillonella atypica*	1
*Escherichia coli*	3	*Pseudomonas aeruginosa*	1
*Streptococcus pneumoniae*	2	*Clostridium perfrigens*	1
*Methicillin-resistant coagulase negative Staphylococci*	2	*Streptococcus gallolyticus subsp. pasteurianus*	1
*Staphylococcus aureus*	2	*Staphylococcus lugdunensis*	1
*Klebsiella variicola*	1	*Enterobacter cloacae complex*	1
*Klebsiella earogenes*	1	*Enterococcus casseliflavus*	1
*Enterococcus faecium*	1	*Candida albicans*	1
*Elizabethkingia meningoseptica*	1		

The total number of infections and pathogens exceeds the number of patients, as some individuals experienced more than one type of infection and multiple pathogens were sometimes isolated from a single infectious episode

**Table 3:** Causative pathogens of pneumonia

Variable	Value	Variable	Value
*Klebsiella pneumoniae*	5	*Schizophyllum commune*	1
*Candida albicans*	4	*Cryptococcus neoformans*	1
*Pseudomonas aeruginosa*	3	*Enterococcus raffinosus*	1
*MRSA (methicillin-resistant Staphylococcus aureus)*	3	*Haemophilus influenzae*	1
*Enterococcus faecium*	2	*Serratia marcescens*	1
*Staphylococcus aureus*	2	*Nocardia cyriacigeorgica*	1
*Corynebacterium striatum*	3	*Klebsiella oxytoca*	1
*Escherichia coli*	2	*Acinetobacter baumannii complex*	1
*Moraxella catarrhalis*	2	*Proteus mirabilis*	1
*BLNAR (β-lactamase-negative ampicillin-resistant Haemophilus influenzae)*	2	*Enterococcus avium*	1
*MRCNS (Methicillin-resistant coagulase-negative staphylococci)*	2	*Enterococcus faecalis*	1
*Acinetobacter baumannii*	2	*Candida parapsilosis*	1
*Elizabethkingia meningoseptica*	1	*Candida lusitaniae*	1
*Stenotrophomonas maltophilia*	1	*Candida glabrata*	1
*Streptococcus agalactiae*	1		

The total number of infections and pathogens exceeds the number of patients, as some individuals experienced more than one type of infection and multiple pathogens were sometimes isolated from a single infectious episode

**Table 4:** Causative pathogens of urinary tract infection

Variable	Value
*Escherichia coli*	5
*Enterococcus faecalis*	5
*Candida albicans*	4
*Corynebacterium striatum*	4
*Klebsiella pneumoniae*	3
*MRSA (methicillin-resistant Staphylococcus aureus)*	2
*Proteus mirabilis*	2
*Pseudomonas aeruginosa*	1
*Veillonella parvula*	1
*Streptococcus anginosus*	1
*MRCNS (Methicillin-resistant coagulase-negative staphylococci)*	1
*Pseudomonas otitidis*	1
*Klebsiella oxytoca*	1
*Candida glabrata*	1
*Streptococcus agalactiae*	1

The total number of infections and pathogens exceeds the number of patients, as some individuals experienced more than one type of infection and multiple pathogens were sometimes isolated from a single infectious episode

### Results of statistical analysis

**Fig. 4:**
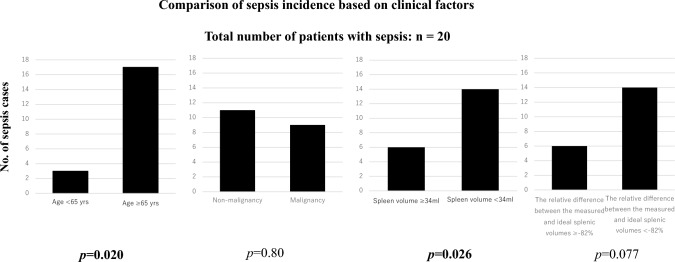
Comparison of sepsis incidence based on clinical factors. Univariate analysis revealed that patients ≥ 65 years were significantly more likely to develop sepsis than those < 65 years (*p* = 0.020). A spleen volume < 34 mL was significantly associated with sepsis (*p* = 0.026). A relative difference between measured and ideal splenic volumes (< −82%) showed a trend toward association with sepsis but was not statistically significant (*p* = 0.077). This figure includes 20 patients who developed sepsis (*n* = 20)

Univariate analysis revealed that patients ≥ 65 years were significantly more likely to develop sepsis than those < 65 years (*p* = 0.020). Additionally, a spleen volume < 34 mL was significantly associated with sepsis (*p* = 0.026). The relative difference between the measured and ideal splenic volumes (< −82%) showed a trend toward association with sepsis, although this was not statistically significant (*p* = 0.077) (Table 5, Fig. 4).

**Table 5:** Infection incidence and clinical factors: univariate analysis

Variable	Non sepsis	Sepsis	*p *value	Non pneumonia	Pneumonia	*p *value	Non UTI	UTI	*p *value	Non infection	At least one infection	*p *value
Age < 65 yrs	33	3	**0.020**	30	6	0.15	31	5	0.30	25	11	**0.011**
Age ≥ 65 yrs	42	17		41	18		45	14		24	35	
History of no malignancy	38	11	0.80	36	13	0.82	40	9	0.80	23	26	0.41
History of malignancy	37	9		35	11		36	10		26	20	
Spleen volume ≥ 34 ml	44	6	**0.026**	41	9	0.10	44	6	0.070	31	19	**0.041**
Spleen volume < 34 ml	31	14		30	15		32	13		18	27	
The relative difference between the measured and ideal splenic volumes												
≥ −82%	41	6	0.077	37	10	0.48	40	7	0.30	27	20	0.31
< −82%	34	14		34	14		36	12		22	26	

Bold indicate statistically significant differences (*p* 0.05)

**Fig. 5:**
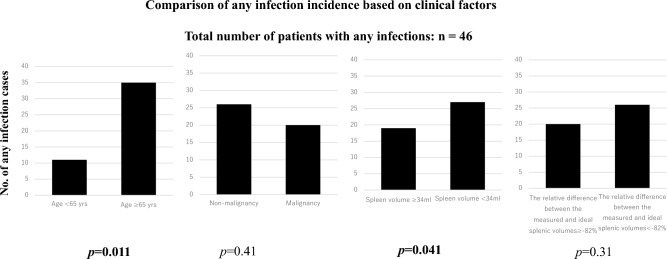
Comparison of any infection incidence based on clinical factors. Univariate analysis revealed that patients ≥ 65 years were significantly more likely to experience at least one infection (*p* = 0.011). Spleen volume < 34 mL was also significantly associated with infection (*p* = 0.041). This figure includes 46 patients who experienced at least one infection (*n* = 46)

Regarding other infection types, no clinical factors were significantly associated with pneumonia or UTIs. However, patients ≥ 65 years were significantly more likely to experience at least one infection (*p* = 0.011), and a spleen volume < 34 mL was also significantly associated with infection occurrence (*p* = 0.041) (Fig. 5).

In the multivariate logistic regression analysis, age ≥ 65 years remained an independent risk factor for sepsis (odds ratio [OR], 4.0; 95% confidence interval [CI] 1.1–15; *p* = 0.039), as did spleen volume < 34 mL (OR, 3.0; 95% CI 1.0–8.9; *p* = 0.047).

Age ≥ 65 years was also significantly associated with the development of at least one infection (OR, 3.1; 95% CI 1.3–7.6; *p* = 0.013). Spleen volume < 34 mL showed a trend toward association but did not reach statistical significance (OR, 2.2; 95% CI 0.96–5.3; *p* = 0.064) (Table 6).

**Table 6:** Sepsis incidence and clinical factors: multiple logistic regression model

Variable	Sepsis		
	OR	95% CI	*p *value
Age (< 65 vs. ≥ 65 yrs)	4.0	1.1–15	**0.039**
Spleen volume (< 34 vs. ≥ 34 ml)	3.0	1.0–8.9	**0.047**

	At least one infection		
	OR	95% CI	*p *value
Age (< 65 vs. ≥ 65 yrs)	3.1	1.3–7.6	**0.013**
Spleen volume (< 34 vs. ≥ 34 ml)	2.2	0.96–5.3	0.064

Bold indicate statistically significant differences (*p* 0.05)

## Discussion

In this study, infection risk in non-splenectomized patients exhibiting HJBs was investigated, revealing that older age (≥ 65 years) and reduced splenic volume (< 34 mL) independently increased susceptibility to sepsis. Although a significantly smaller actual-to-ideal splenic volume ratio (≤ 82%) showed a trend toward sepsis risk, this difference was not statistically significant. These findings suggest that a spleen volume threshold of 34 mL may be clinically meaningful for identifying patients at higher risk of severe infections, particularly sepsis. Moreover, older age emerged as a robust predictor of infection. This highlights the potential value of targeted prophylactic measures—such as vaccinations and antibiotic therapy—in patients with suspected functional hyposplenism.

The precise mechanisms underlying splenic atrophy in functional hyposplenism remain poorly understood. Picardi et al*.*[14] hypothesized that immune-mediated processes similar to those causing pancreatic atrophy in diabetes may underlie progressive splenic shrinkage. Supporting this immunological perspective, the study shows that patients with autoimmune pancreatitis initially presented with splenomegaly, which later progressed to splenic atrophy after steroid treatment [15]. The proposed mechanism involves the expansion of autoreactive lymphocytes that deplete naive lymphocyte populations, ultimately reducing spleen volume. Similarly, experimental studies in murine models show that persistent antigen presentation can induce splenic atrophy [16]. Consistent with these observations, a case of prolonged chronic graft-versus-host disease (GVHD) is associated with ongoing splenic atrophy [5], further supporting an immune-mediated etiology. Although these findings strongly suggest an immunological basis, further research is warranted to elucidate the precise biological pathways involved.

HJBs are well-recognized markers of impaired splenic function under standard light microscopy [3–6]. Although HJBs’ sensitivity and specificity may be lower than those of more specialized tests—such as radioisotope scanning, flow cytometry for IgM^+^ CD27^+^ B cells, or pitted erythrocyte counts [6, 17–20]—their simplicity and low cost make them a practical screening method across diverse clinical settings. Patients with detectable HJBs could undergo additional confirmatory testing or closer clinical monitoring, especially if imaging also reveals reduced splenic volume. Integrating HJBs detection with imaging findings enhances the ability of clinicians to identify individuals at risk for functional hyposplenism.

CT–based measurement of the splenic volume provides greater precision than traditional single-dimensional assessments (e.g., measuring the splenic long axis) [12, 15]. Although splenomegaly is commonly reported in radiologic assessment, splenic atrophy is often overlooked or underreported [21]. Although indices such as the spleen index are used to evaluate splenomegaly, statistical reports on splenic atrophy remain limited, and no clear criteria have been established [22]. Whereas Picardi et al. [14] identified a two-dimensional splenic index using ultrasound, our CT-derived volumetric cut-off provides a three-dimensional, operator-independent assessment that can be applied to routine abdominal CT scans, thereby enhancing the clinical utility of imaging-based evaluation for functional hyposplenism. Nevertheless, reduced splenic volume alone does not definitively diagnose functional hyposplenism. Radioisotope-based assays and specialized immunological tests remain the gold standard for confirming true splenic dysfunction [23, 24]. However, these modalities are expensive, require specialized equipment, and may not be readily available in all healthcare settings. Consequently, CT-based spleen volume measurements serve as an accessible and relatively accurate indicator of potential functional hyposplenism—especially when interpreted alongside HJB status [5, 6].

The spleen plays a multifaceted role in immune defense, contributing to antigen presentation, antibody production, phagocytosis, and opsonization of blood-borne pathogens [1]. A previous study [25] shows that functional hyposplenism increases the risk of infection, particularly sepsis. However, quantifying this risk relative to that of splenectomized patients remains challenging, given limited comparative data [26]. In splenectomized individuals, the risk of overwhelming post-splenectomy infection (OPSI) is > 50 times that of the general population, with *S. pneumoniae* as the predominant causative pathogen (50–90% of cases), followed by *H. influenzae* type b and *N. meningitidis* [27]. Functional hyposplenism may confer similar susceptibility; however, direct comparative evidence is lacking [26]. The microorganisms most commonly responsible for OPSI include *Streptococcus pneumoniae*, *Haemophilus influenzae* type b, and *N. meningitidis*, with less frequently reports on *Klebsiella pneumoniae* and *Salmonella typhi*, among others [7]. Therefore, a similar distribution of pathogens was expected in our cohort of patients with functional hyposplenism. However, *Klebsiella pneumoniae* emerged as the most frequently detected organism. This unexpected predominance may reflect the widespread use of effective vaccines against *Streptococcus pneumoniae*, *Haemophilus influenzae* type b, and *N. meningitides*, but no commercially available vaccine currently exists for *Klebsiella pneumoniae*.

Given this vulnerability, clinical guidelines have increasingly shifted to treating patients with functional hyposplenism similar to those who have undergone splenectomy. In particular, the latest British guidelines^17^, which reference our single-center research on HJBs detection [6], emphasize that both splenectomized and functionally hyposplenic patients require vaccination against *S. pneumoniae*, *H. influenzae* type b, and *N. meningitidis*. Moreover, lifelong prophylactic antibiotics therapy is recommended for individuals at high risk of pneumococcal infection—especially young children (< 16 years), older adults (> 50 years), patients with poor serological responses, or those with a history of invasive pneumococcal disease. Other high-risk populations include patients with hematological malignancies—particularly those undergoing splenic irradiation, experiencing GVHD, or long-term immunosuppression—as well as those in the early postoperative period following post-splenectomy (within 1–3 years). These recommendations highlight the significance of accurately identifying patients with potential splenic dysfunction, whether anatomical or functional.

Our findings support this approach. Through the establishment of a specific spleen volume threshold (< 34 mL) associated with increased sepsis risk and identifying older age as an additional key predictor, we contribute further details to the existing evidence base. These findings may help clinicians in personalizing preventive strategies, including timely vaccination and antibiotic prophylaxis. Nonetheless, larger prospective studies are warranted to determine the optimal frequency and methodology for monitoring splenic volume. They are also needed to refine prophylactic approaches (e.g., duration of antibiotic use) and to directly compare outcomes between patients with functional hyposplenism and those who have undergone splenectomy.

Despite these findings, this study has some limitations. First, the single-center, retrospective design may limit generalizability, and reliance on culture-positive cases may underestimate the incidence of infection. Second, incomplete data on vaccination status and antibiotic prophylaxis hindered a thorough evaluation of their protective effects. Third, since many patients underwent CT imaging after an infectious event, time-dependent sepsis risks could not be calculated. Finally, although HJB detection is a practical screening tool for splenic dysfunction, its diagnostic accuracy is inferior to that of more specialized methods. Larger prospective studies—particularly those incorporating etiologically stratified cohorts—are required to determine whether specific patient subgroups exhibit distinct infection profiles or require more intensive prophylaxis. A lack of microbiological data meant that subgroup analyses of causative organisms by age or spleen volume could not be conducted in this study. Future studies with larger sample sizes are needed to explore whether specific pathogens are more prevalent in specific subgroups of patients. While CT-based spleen volumetric measurement provides precise splenic function assessment, repeated imaging poses cost and resource challenges. Therefore, further research into alternative or targeted screening protocols may help balance the feasibility and clinical benefits.

Older age (≥ 65 years) and reduced splenic volume (< 34 mL) independently predict sepsis risk in non-splenectomized patients with HJBs. These findings underscore the need to carefully evaluate splenic volume alongside HJBs detection to identify and manage functional hyposplenism. Future prospective investigations should aim to refine prophylactic strategies, establish optimal screening intervals, deepen understanding of the underlying mechanisms, and ultimately improve outcomes in this at-risk population.

## Data Availability

The datasets generated and analyzed in this study are not publicly available owing to patient privacy concerns but can be obtained from the corresponding author on reasonable request.

## References

[1] Mebius RE, Kraal G. Structure and function of the spleen. Nat Rev Immunol. 2005;5(8):606–16. 16056254 10.1038/nri1669

[2] Cooke KR, Luznik L, Sarantopoulos S, Hakim FT, Jagasia M, Fowler DH, et al. The biology of chronic graft-versus-host disease: a task force report from the National Institutes of Health consensus development project on criteria for clinical trials in chronic graft-versus-host disease. Biol Blood Marrow Transplant. 2017;23(2):211–34. 27713092 10.1016/j.bbmt.2016.09.023 PMC6020045

[3] Dameshek W. Hyposplenism. JAMA. 1955;157(7):613.

[4] Pearson HA, Johnston D, Smith KA, Touloukian RJ. The born-again spleen. Return of splenic function after splenectomy for trauma. N Engl J Med. 1978;298(25):1389–92. 652006 10.1056/NEJM197806222982504

[5] Uchino K, Nakagami Y, Enotomoto M, Shimizu N, Kondo K, Yamamoto T, et al. Underrecognised functional hyposplenism associated with chronic graft-versus-host disease: a case report. EJHaem. 2025;6(2): e70017. 40078599 10.1002/jha2.70017 PMC11902891

[6] Nakagami Y, Uchino K, Okada H, Suzuki K, Enomoto M, Mizuno S, et al. Potential role of Howell-Jolly bodies in identifying functional hyposplenism: a prospective single-institute study. Int J Hematol. 2020;112(4):544–52. 32572828 10.1007/s12185-020-02925-7

[7] Kirkineska L, Perifanis V, Vasiliadis T. Functional hyposplenism. Hippokratia. 2014;18(1):7–11. 25125944 PMC4103047

[8] William BM, Corazza GR. Hyposplenism: a comprehensive review. Part I: basic concepts and causes. Hematology. 2007;12(1):1–13. 17364987 10.1080/10245330600938422

[9] Sugawara Y, Hayashi Y, Shigemasa Y, Abe Y, Ohgushi I, Ueno E, et al. Molecular biosensing mechanisms in the spleen for the removal of aged and damaged red cells from the blood circulation. Sensors (Basel). 2010;10(8):7099–121. 22163593 10.3390/s100807099 PMC3231191

[10] Corazza GR, Ginaldi L, Zoli G, Frisoni M, Lalli G, Gasbarrini G, et al. Howell-jolly body counting as a measure of splenic function. A reassessment. Clin Lab Haematol. 1990;12(3):269–75. 2125541 10.1111/j.1365-2257.1990.tb00037.x

[11] Inaba T, Ohama A. Prominent increase of Pappenheimer body-containing erythrocytes in a patient with hypoplastic spleen (IJHM-D-18-00279R2). Int J Hematol. 2018;108(4):351–2. 30083850 10.1007/s12185-018-2512-5

[12] Harris A, Kamishima T, Hao HY, Kato F, Omatsu T, Onodera Y, et al. Splenic volume measurements on computed tomography utilizing automatically contouring software and its relationship with age, gender, and anthropometric parameters. Eur J Radiol. 2010;75(1):e97-101. 19775843 10.1016/j.ejrad.2009.08.013

[13] Kanda Y. Investigation of the freely available easy-to-use software “EZR” for medical statistics. Bone Marrow Transplant. 2013;48(3):452–8. 23208313 10.1038/bmt.2012.244 PMC3590441

[14] Picardi M, Selleri C, Rotoli B. Spleen sizing by ultrasound scan and risk of pneumococcal infection in patients with chronic GVHD: preliminary observations. Bone Marrow Transplant. 1999;24(2):173–7. 10455346 10.1038/sj.bmt.1701861

[15] Matsubayashi H, Uesaka K, Kanemoto H, Aramaki T, Nakaya Y, Kakushima N, et al. Reduction of splenic volume by steroid therapy in cases with autoimmune pancreatitis. J Gastroenterol. 2013;48(8):942–50. 23076542 10.1007/s00535-012-0692-y

[16] Mbanwi AN, Wang C, Geddes K, Philpott DJ, Watts TH. Irreversible splenic atrophy following chronic LCMV infection is associated with compromised immunity in mice. Eur J Immunol. 2017;47(1):94–106. 27730627 10.1002/eji.201646666

[17] Ladhani SN, Fernandes S, Garg M, Borrow R, de Lusignan S, Bolton-Maggs PHB, et al. Prevention and treatment of infection in patients with an absent or hypofunctional spleen: a British Society for Haematology guideline. Br J Haematol. 2024;204(5):1672–86. 38600782 10.1111/bjh.19361

[18] Lammers AJ, de Porto AP, Bennink RJ, van Leeuwen EM, Biemond BJ, Goslings JC, et al. Hyposplenism: comparison of different methods for determining splenic function. Am J Hematol. 2012;87(5):484–9. 22488175 10.1002/ajh.23154

[19] Sills RH. Splenic function: physiology and splenic hypofunction. Crit Rev Oncol Hematol. 1987;7(1):1–36. 3304675 10.1016/s1040-8428(87)80012-4

[20] Spencer RP, Gupta SM. The spleen: diagnosis of splenic diseases using radiolabeled tracers. Crit Rev Clin Lab Sci. 1989;27(4):299–318. 2675910 10.3109/10408368909105717

[21] Vancauwenberghe T, Snoeckx A, Vanbeckevoort D, Dymarkowski S, Vanhoenacker FM. Imaging of the spleen: what the clinician needs to know. Singapore Med J. 2015;56(3):133–44. 25820845 10.11622/smedj.2015040 PMC4371192

[22] Linguraru MG, Sandberg JK, Jones EC, Summers RM. Assessing splenomegaly: automated volumetric analysis of the spleen. Acad Radiol. 2013;20(6):675–84. 23535191 10.1016/j.acra.2013.01.011 PMC3945039

[23] Demetrakopoulos GE, Tsokos GC, Levine AS. Recovery of splenic function after GVHD-associated functional asplenia. Am J Hematol. 1982;12(1):77–80. 7039310 10.1002/ajh.2830120112

[24] Au WY, Ma SK, Wong KK. Hyposplenism after allogeneic bone marrow transplantation. Br J Haematol. 2002;117(3):488. 12028014 10.1046/j.1365-2141.2002.03514.x

[25] William BM, Thawani N, Sae-Tia S, Corazza GR. Hyposplenism: a comprehensive review. Part II: clinical manifestations, diagnosis, and management. Hematology. 2007;12(2):89–98. 17454188 10.1080/10245330600938463

[26] Di Sabatino A, Carsetti R, Corazza GR. Post-splenectomy and hyposplenic states. Lancet. 2011;378(9785):86–97. 21474172 10.1016/S0140-6736(10)61493-6

[27] Hansen K, Singer DB. Asplenic-hyposplenic overwhelming sepsis: postsplenectomy sepsis revisited. Pediatr Dev Pathol. 2001;4(2):105–21. 11178626 10.1007/s100240010145

